# Clinical Characteristics of 26 Human Cases of Highly Pathogenic Avian Influenza A (H5N1) Virus Infection in China

**DOI:** 10.1371/journal.pone.0002985

**Published:** 2008-08-21

**Authors:** Hongjie Yu, Zhancheng Gao, Zijian Feng, Yuelong Shu, Nijuan Xiang, Lei Zhou, Yang Huai, Luzhao Feng, Zhibin Peng, Zhongjie Li, Cuiling Xu, Junhua Li, Chengping Hu, Qun Li, Xiaoling Xu, Xuecheng Liu, Zigui Liu, Longshan Xu, Yusheng Chen, Huiming Luo, Liping Wei, Xianfeng Zhang, Jianbao Xin, Junqiao Guo, Qiuyue Wang, Zhengan Yuan, Longnv Zhou, Kunzhao Zhang, Wei Zhang, Jinye Yang, Xiaoning Zhong, Shichang Xia, Lanjuan Li, Jinquan Cheng, Erdang Ma, Pingping He, Shui Shan Lee, Yu Wang, Timothy M. Uyeki, Weizhong Yang

**Affiliations:** 1 Office for Disease Control and Emergency Response, Chinese Center for Disease Control and Prevention (China CDC), Beijing, China; 2 Department of Respiratory Medicine, Peking University People's Hospital, Beijing, China; 3 State Key Laboratory for Infectious Disease Prevention and Control, National Institute for Viral Disease Control and Prevention, China CDC, Beijing, China; 4 Hunan Provincial Center for Disease Control and Prevention, Changsha, China; 5 Xiang Ya Hospital of Central South University, Changsha, China; 6 Anhui Provincial Center for Disease Control and Prevention, Hefei, China; 7 Anhui Provincial Hospital, Hefei, China; 8 Sichuan Provincial Center for Disease Control and Prevention, Chengdu, China; 9 Huaxi Hospital, Sichuan University, Chengdu, China; 10 Fujian Provincial Center for Disease Control and Prevention, Fuzhou, China; 11 Fujian Provincial Hospital, Fuzhou, China; 12 Guangdong Provincial Center for Disease Control and Prevention, Guangzhou, China; 13 Third Affiliated Hospital, Guangzhou Medical College, Guangzhou, China; 14 Hubei Provincial Center for Disease Control and Prevention, Wuhan, China; 15 Hankou Union Hospital, Hubei Province, Wuhan, China; 16 Liaoning Provincial Center for Disease Control and Prevention, Shenyang, China; 17 First Affiliated Hospital, China Medical University, Shenyang, China; 18 Shanghai Center for Disease Control and Prevention, Shanghai, China; 19 Ninth Affiliated Hospital, Shanghai Transportation University, Shanghai, China; 20 Jiangxi Provincial Center for Disease Control and Prevention, Nanchang, China; 21 First Affiliated Hospital, Nanchang University, Nanchang, China; 22 Guangxi Provincial Center for Disease Control and Prevention, Nanning, China; 23 First Affiliated Hospital, Guangxi Medical University, Nanning, China; 24 Zhejiang Provincial Center for Disease Control and Prevention, Hangzhou, China; 25 First Affiliated Hospital, Zhejiang University, Hangzhou, China; 26 Shenzhen Center for Disease Control and Prevention, Shenzhen, China; 27 Xinjiang Uygur Autonomous Region Center for Disease Control and Prevention, Urumqi, China; 28 Department of Epidemiology and Biostatistics School of Public Health, Health Science Center, Peking University, Beijing, China; 29 Centre for Emerging Infectious Diseases, Chinese University of Hong Kong, Hong Kong Special Administrative Region, China; 30 Influenza Division, National Center for Immunization and Respiratory Diseases, Centers for Disease Control and Prevention, Atlanta, Georgia, United States of America; U.S. Naval Medical Research Center Detachment/Centers for Disease Control, United States of America

## Abstract

**Background:**

While human cases of highly pathogenic avian influenza A (H5N1) virus infection continue to increase globally, available clinical data on H5N1 cases are limited. We conducted a retrospective study of 26 confirmed human H5N1 cases identified through surveillance in China from October 2005 through April 2008.

**Methodology/Principal Findings:**

Data were collected from hospital medical records of H5N1 cases and analyzed. The median age was 29 years (range 6–62) and 58% were female. Many H5N1 cases reported fever (92%) and cough (58%) at illness onset, and had lower respiratory findings of tachypnea and dyspnea at admission. All cases progressed rapidly to bilateral pneumonia. Clinical complications included acute respiratory distress syndrome (ARDS, 81%), cardiac failure (50%), elevated aminotransaminases (43%), and renal dysfunction (17%). Fatal cases had a lower median nadir platelet count (64.5×10^9^ cells/L *vs* 93.0×10^9^ cells/L, p = 0.02), higher median peak lactic dehydrogenase (LDH) level (1982.5 U/L *vs* 1230.0 U/L, p = 0.001), higher percentage of ARDS (94% [n = 16] *vs* 56% [n = 5], p = 0.034) and more frequent cardiac failure (71% [n = 12] *vs* 11% [n = 1], p = 0.011) than nonfatal cases. A higher proportion of patients who received antiviral drugs survived compared to untreated (67% [8/12] *vs* 7% [1/14], p = 0.003).

**Conclusions/Significance:**

The clinical course of Chinese H5N1 cases is characterized by fever and cough initially, with rapid progression to lower respiratory disease. Decreased platelet count, elevated LDH level, ARDS and cardiac failure were associated with fatal outcomes. Clinical management of H5N1 cases should be standardized in China to include early antiviral treatment for suspected H5N1 cases.

## Introduction

As of July 13, 2008, 385 confirmed human cases of infection with highly pathogenic avian influenza A (H5N1) virus with 243 deaths had been reported from 15 countries since November, 2003 [1]. Although largely a panzoonotic among poultry and wild birds, avian-to-human transmission of H5N1 virus has resulted in most human cases [2], with rare instances of limited, non-sustained human-to-human H5N1 virus transmission [3]–[5]. The continuing propagation of highly pathogenic H5N1 viruses among poultry [6] and migratory birds [7], [8] poses a continuing and potentially escalating threat to human populations. Preparedness for a possible H5N1 pandemic requires not only enhanced prevention efforts but also a heightened awareness of the clinical characteristics of H5N1 cases among physicians.

To date, limited H5N1 clinical data are available in case reports and limited case series from Hong Kong Special Administrative Region (SAR), China in 1997 [9] and 2003 [10], and Vietnam [11], [12], Thailand [13]–[15], Indonesia [4], [16], Cambodia [17], Azerbaijan [18], [19], and Turkey [20] during 2004–2006. These observational studies described symptoms, signs, and laboratory findings at hospital admission. Few data are available on the clinical characteristics of cases throughout the course of H5N1 disease. Data on the natural history of H5N1 disease may allow risk stratification and identification of prognostic factors for outcomes of H5N1 virus infection. We describe the natural history and report the clinical characteristics at illness onset, hospital admission, and throughout hospitalization for 26 H5N1 cases identified by surveillance between October 2005 and April 2008.

## Methods

### National surveillance system and case definitions

In China, all suspected H5N1 cases are reported to the Chinese Center for Disease Control and Prevention (China CDC, Beijing, China) through a national surveillance system, which is based upon reporting of hospitalized cases of pneumonia of unknown origin, and by enhanced 1-month surveillance for cases of influenza-like illness at all health-care facilities within a 3-km radius after the occurrence of a suspected or confirmed H5N1 poultry outbreak with high bird mortality.

A case of pneumonia of unknown origin was defined as a patient with all of the following criteria without specific laboratory diagnosis: fever (temperature ≥38°C); radiological evidence of pneumonia or acute respiratory distress syndrome (ARDS); normal white blood cell count (WBC; range 4–10×10^9^ cells per L), leukopenia (WBC <4×10^9^ cells per L), or lymphopenia (lymphocyte count <0.8×10^9^ cells per L) at clinical presentation; and absence of clinical improvement after treatment with broad-spectrum antibiotics. A case of influenza-like illness was defined as a patient with fever (temperature ≥38°C) and cough or sore throat, in the absence of any other confirmed diagnosis.

A confirmed case of H5N1 was defined as a patient with pneumonia or influenza-like illness and laboratory evidence of H5N1 virus infection diagnosed by viral isolation or reverse transcriptase (RT) PCR by testing respiratory specimens, or a four-fold or greater increase in H5N1 antibody titre in paired acute and convalescent sera.

### Case-patients

All suspected H5N1 case-patients were interviewed by staff of the local CDC, and respiratory specimens, and acute- and convalescent-phase sera were obtained if available for laboratory investigations following the WHO protocol [21]. Respiratory specimens were tested by conventional [22] and real-time RT-PCR [23] to detect H5-specific viral RNA in biosafety level (BSL) 2 facilities at the National Influenza Center (NIC) of China CDC, and were inoculated into amniotic and/or allantoic cavities of specific pathogen free (SPF) embryonated chicken eggs for viral isolation [24] in enhanced BSL 3 facilities at the NIC. H5N1 antibody testing was performed on sera at the NIC by microneutralization (MN) assay [25] in a BSL-3 laboratory, and modified hemagglutination-inhibition (HI) assay using horse red blood cells [26] in BSL-2 conditions.

In mainland China, 30 confirmed human H5N1 cases have been identified to date. We included 26 laboratory-confirmed H5N1 cases identified by surveillance in 12 provinces in China between October 2005 and April 2008. Our analyses included limited data from 2 case reports [27], [28] and 6 urban cases reported previously in a brief epidemiological dispatch [29]. Of the 26 cases, H5N1 virus infection was confirmed by both virus isolation and RT-PCR in 20 (77%) cases, one (4%) case by virus isolation only, three (11%) by RT-PCR and serology, and two (8%) by serology only. Twenty-four cases in southern China were infected with clade 2.3.4 H5N1 viruses, and two cases from northern China had clade 2.2 H5N1 virus infections [2]. We excluded four H5N1 cases, including 2 military cases with unavailable clinical data and 2 cases in a cluster with limited person-to-person transmission reported elsewhere [5].

### Clinical investigations

A trained team from the China CDC interviewed all confirmed H5N1 cases or their proxies, and collected clinical data through review of hospital medical records. A standardized form was used to collect information on demographic characteristics and clinical data, including clinical findings, blood chemistry testing and chest radiograph results performed during clinical management, complications, treatments, and outcomes. Data were collected during field investigations by China CDC staff, and was part of a continuing public-health outbreak investigation and determined by the Ministry of Health to be exempt from institutional review board assessment in China.

We used the following definitions: cardiac failure was defined as requiring use of inotropic agents; respiratory failure was defined as the need for assisted ventilatiory support; ARDS was defined as clinical deterioration with severe arterial hypoxaemia and diffuse bilateral infiltrates on chest radiograph; disseminated intravenous coagulation (DIC) was defined as elevated prothrombin time (PT) with elevated activated partial thromboplastin time (APTT), and decreased fibrinogen (FIB) level with thrombocytopenia; liver function impairment was defined as aminotransferase (ALT or AST) levels ≥ 2× upper range of normal values; renal dysfunction was defined as creatinine level >178mmol/L for adults or ≥ 2× upper limit of normal for age. High-dose corticosteroid use was defined as ≥250 mg hydrocortisone or equivalent intravenous (IV) administration daily. For children <13 years old, high-dose corticosteroid use was defined as ≥5 mg hydrocortisone or equivalent IV/kg/day.

### Statistical analysis

Medians and interquartile ranges (IQRs) were calculated for continuous variables, and compared between fatal and nonfatal cases using Wilcoxon rank sum test. For categorical variables, percentages of case-patients in each category were compared using Fisher's exact test. Fatal cases were compared to nonfatal cases by demographic characteristics, H5N1 virus clade, underlying medical conditions, medical care practices, haematological and biochemical markers at admission or during hospitalization, clinical complications, and treatments, in the bivariate analyses using logistic regression. All statistical tests were two-sided with a significance level set at *α* = 0.05. Data were analyzed with SPSS (version 13.0, SPSS Inc, Chicago, IL, USA).

## Results

Twenty-six confirmed H5N1 cases had illness onset beginning in October, 2005 through February 2008. The median age of the 26 cases was 29 years (range 6–62) and 58% were female. Five (19%) were children aged <10 years old, one (4%) was 16 years old, and 20 (77%) were adults aged >18 years.

### Clinical presentation

The earliest reported symptoms and signs of 26 patients at illness onset and noted at hospital admission are shown in Table 1. Many patients reported fever (92%) or cough (58%) initially, but very few reported upper respiratory symptoms such as rhinorrhea or sore throat. All patients developed cough a median of 1 day (IQR 1–3) from illness onset, and 85% had sputum production a median of 3 days (IQR 1–5.3) after illness onset. Lower respiratory tract signs and symptoms such as tachypnea and dyspnea increased substantially from illness onset to hospital admission. Most patients (88%) had tachypnea a median of 5 days (IQR 4–7) from illness onset and 46% reported dyspnea a median of 6.5 days (IQR 4.5–8.5) from illness onset. Diarrhea was reported in only one adult case at illness onset, and in two cases at hospital admission, but developed in six patients (one child and five adults) after hospitalization. The duration of diarrhea in these nine (35%) cases was a median of 1 day (IQR 1–4).

**Table 1:** Signs and symptoms of 26 H5N1 cases at illness onset and at hospital admission, China.

Signs and symptoms	At illness onset, no. (%)	At hospital admission, no. (%)
Fever	24 (92)	18 (69)
Chills	12 (46)	12 (46)
Malaise	10 (39)	12 (46)
Myalgia	11 (42)	8 (31)
Headache	4 (15)	4 (15)
Sore throat	2 (8)	4 (15)
Rhinorrhea	3 (12)	1 (4)
Cough	15 (58)	20 (77)
Sputum production	9 (35)	15 (58)
Tachypnea*	2 (8)	18 (69)
Dyspnea	0	6 (23)
Vomiting	2 (8)	3 (12)
Abdominal pain	1 (4)	1 (4)
Diarrhea	1 (4)	2 (8)

* Respiratory rate >24 per min.

**Figure 1:**
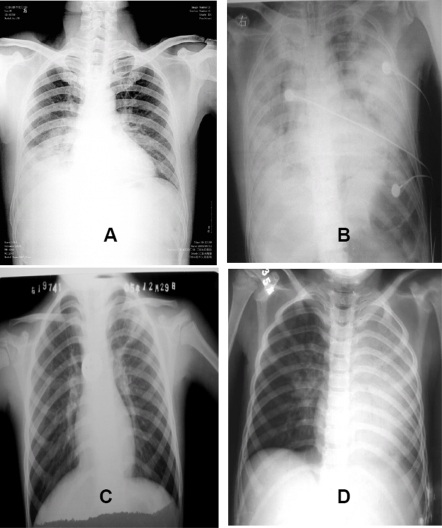
Progression of pulmonary disease in chest radiographs from adult (35-year-old male, Panels A day 6 and B day 23 of illness) and pediatric (6-year-old male, Panels C day 6 and D day 14 of illness) H5N1 cases.

All case-patients had abnormal chest radiographs at admission; unilateral or bilateral infiltrates were observed in 10 (38%) and 16 (62%) case-patients, at a median of 6.5 days (IQR 4–7.3) and 7.5 days (IQR 6.3–9) from illness onset, respectively. The 10 case-patients with unilateral infiltrates at admission all developed bilateral pneumonia (Table 2). Chest radiographs showing rapid progression from unilateral to bilateral pulmonary infiltrates and ARDS in adult and paediatric cases are shown in Figure 1. Radiographic findings included patchy or diffuse infiltrates or consolidation with air bronchograms in multi-segmental or lobular distribution.

**Table 2:** Initial chest radiographic findings and progression during hospitalization of 26 H5N1 cases, China.

Initial radiographic findings	No. (%)	Median days after illness onset (IQR)	Progression during hospitalization	No. (%)	Median days after illness onset (IQR)
Normal	0	0	No change	0	0
			Unilateral infiltrates	0	0
			Bilateral infiltrates	0	0
Unilateral infiltrates	10 (38)	6.5 (4–7.3)	No change	0	0
			Bilateral infiltrates	10 (100)	8 (6.8–9.5)
Bilateral infiltrates	16 (62)	7.5 (6.3–9)	NA*	NA*	NA*

* NA demotes not applicable

### Laboratory findings

Laboratory findings on initial testing, at admission and during hospitalization are shown in Table 3. The prevalence of patients with abnormal haematological findings at admission [leukopenia (46%), lymphopenia (62%), and moderate thrombocytopenia (50%)] increased to 92%, 89% and 73%, respectively, during hospitalization. At admission, the median leukocyte count was 3.5×10^9^ cells/L (IQR 2.3–4.5) and median lymphocyte count was 0.6×10^9^ cells/L (IQR 0.4–1.0). These declined during hospitalization to a median leukocyte count of 2.3×10^9^ cells/L (IQR 1.5–2.8) and median lymphocyte count of 0.3×10^9^ cells/L (IQR 0.3–0.5), after a median of 8.0 days.

**Table 3:** Laboratory findings of 26 H5N1 cases* on initial testing, at hospital admission, and during hospitalization, China.

Variables	Initial test			At hospital admission			Progression (peak or nadir measurement) during hospitalization		
	Median (IQR)	No./Total (%) Abnormal	Median days after illness onset (IQR)	Median (IQR)	No./Total (%) Abnormal	Median days after illness onset (IQR)	Median (IQR)	No./Total (%) Abnormal	Median days after illness onset (IQR)
Haematology†									
WBC	4.3 (2.6–5.9)	12/26 (46)	6.0 (5.0–8.0)	3.5 (2.3–4.5)	18/26 (69)	8.0 (6.0–10.0)	2.3 (1.5–2.8)	25/26 (92)	8.0 (7.0–10.5)
LYM	0.7 (0.5–1.1)	16/26 (62)	7.0 (5.0–9.0)	0.6 (0.4–1.0)	18/26 (69)	8.0 (6.8–10.0)	0.3 (0.3–0.5)	23/26 (89)	8.0 (7.0–12.0)
PLT	109.5 (73.8–146.3)	13/26 (50)	6.0 (5.0–8.3)	86.0 (64.0–122.5)	16/26 (62)	8.0 (6.8–10.0)	67.0 (42.0–86.0)	19/26 (73)	9.0 (8.0–11.0)
Serum biochemistry‡									
ALT	42.5 (23.3–166.3)	13/24 (54)	8.0 (7.0–10.0)	50.5 (20.8–166.3)	14/24 (58)	8.0 (7.0–10.0)	183.0 (103.0–230.0)	19/24 (79)	12.0 (8.0–16.0)
AST	157.5 (66.0–472.1)	23/24 (89)	8.0 (7.0–10.0)	166.5 (64.5–487.8)	23/24 (89)	8.5 (7.0–10.0)	276.5 (129.0–682.3)	24/24 (100)	10.0 (8.0–11.8)
Albumin	29.8 (27.1–34.1)	17/22 (77)	8.0 (6.8–10.0)	29.8 (27.1–34.1)	17/22 (77)	8.0 (6.8–10.0)	27.8 (25.0–30.2)	21/22 (95)	9.0 (7.0–10.0)
Creatinine	75.5 (57.1–106.5)	4/24 (17)	8.0 (7.0–10.0)	73.0 (60.3–110.0)	5/23 (22)	8.0 (7.0–10.0)	243.2 (157.3–362.3)	6/24 (25)	11.0 (8.5–24.0)
CK	801.0 (162.3–1268.3)	15/20 (75)	8.0 (7.0–10.0)	901.5 (261.3–1420.2)	16/20 (80)	9.0 (7.0–11.0)	1293.0 (658.0–6545.0)	19/20 (95)	10.0 (9.0–12.0)
CK-MB	37.5 (16.0–86.0)	12/17 (71)	8.0 (7.0–10.5)	42.0 (16.0–86.0)	12/17 (71)	9.0 (7.0–11.0)	78.5 (68.3–241.2)	14/17 (82)	11.0 (8.0–14.3)
LDH	1141.0 (634.0–1770.0)	20/21 (95)	8.0 (7.0–10.5)	1298.0 (753.5–1899.0)	20/21 (95)	9.0 (7.0–11.0)	1733 (1218.5–2055.5)	21/21 (100)	10.0 (8.5–11.5)
Plasma glucose concentration	7.9 (6.4–9.9)	18/23 (78)	7.0 (6.0–9.0)	8.2 (6.3–10.1)	17/22 (73)	8.0 (6.0–10.0)	15.6 (10.4–20.6)	21/23 (91)	10.0 (9.0–11.0)
Coagulation parameters#									
PT	13.5 (12.4–15.8)	6/20(30)	10.0 (8.0–11.0)	13.5 (12.4–15.2)	5/18 (28)	10.0 (8.0–11.0)	19.3 (16.9–23.3)	6/20 (30)	10.5 (8.8–11.3)
APTT	35.1 (29.6–49.2)	9/20 (45)	10.0 (8.3–11.8)	35.6 (31.3–48.9)	8/17 (47)	10.0 (8.0–11.5)	51.5 (48.6–54.6)	12/20 (60)	10.0 (8.3–13.8)
FIB	2.3 (1.9–3.0)	2/16 (12.5)	9.0 (8.0–11.0)	2.3 (1.9–3.0)	2/16 (12.5)	9.0 (8.0–11.0)	4.3 (4.1–6.8)	4/16 (25)	8.5 (11.0–14.3)
Urinalysis§									
Proteinuria	NA¶¶	14/22 (64)	8.0 (6.8–10.3)	NA¶¶	14/19 (74)	8.0 (7.0–10.0)	NA¶¶	17/22 (77)	9.0 (7.0–11)
Red blood cell +	NA¶¶	11/20 (55)	8.0 (6.3–10.8)	NA¶¶	12/18 (67)	8.0 (6.8–10.3)	NA¶¶	13/20 (65)	8.0 (7.0–10.5)
Write blood cell +	NA¶¶	2/20 (10)	8.0 (6.0–10.8)	NA¶¶	2/18 (11)	8.0 (6.8–10.3)	NA¶¶	4/20 (20)	8.0 (7.0–9.8)
Urinary casts	NA¶¶	3/10 (30)	9.0 (7.0–12.3)	NA¶¶	2/8 (25)	8.0 (7.0–11.3)	NA¶¶	3/10 (30)	9.0 (7.0–12.3)

* Indicates denominators for testing of fewer cases than full group.

† Abbreviations and normal range: WBC, white blood cell, 4.0–10.0×10^9^ cells per L, leukopenia (abnormal) was defined as leukocyte count less than 4×10^9^ per L; LYM, lymphocyte count, 0.8–4.0×10^9^ cells per L, lymphopenia (abnormal) was defined as lymphocyte count less than 0.8×10^9^ per L; PLT, platelet count, 100–300×10^9^ platelets per L, thrombocytopenia (abnormal) was defined as platelet count less than 100×10^9^ per L.

‡ Abbreviations and normal range: ALT, alanine aminotrasferase, 0.0–45.0 U/L; AST, aspartate aminotransferase, 0–45 U/L; Albumin, 35.0–55.0g/L; Creatinine, 36.0–144.0 µmol/L; CK, creatine kinase, 25–190 U/L, abnormal was defined as >130 IU/L for males and >110 IU/L for females; CK-MB, creatine phosphokinase isoenzymes, 0–25 U/L; LDH, Lactic dehydrogenase, 110–250 U/L; Plasma glucose concentration, 3.33–5.55 mmol/L for <15 years, 3.89–5.83 mmol/L for adults (16–59 years), 4.44–6.38 mmol/L for age >60 years, hyperglycemia (abnormal) was defined as plasma glucose concentration above the upper limit.

# Abbreviations and normal range: PT, prothrombin time, 11–13 second, abnormal was defined as 3 seconds longer than the upper range of normal; APTT, activated partial thromboplastin time, 26–36 second, abnormal was defined as 3 seconds longer than the upper range of normal; FIB, fibrinogen, 2.0–4.0 g/L, abnormal was defined as <2.0 g/L.

§ Normal ranges: total protein (below 120 mg/L); red blood cells (0 to 1 per average high powered field [HPF×400)); white blood cells (1–4 per HPF×400)

¶¶ NA demotes not applicable.

Abnormal percentage, peak measurement and median days from illness onset of biochemical markers on initial testing, at hospital admission, and during hospitalization are shown in Table 3. Elevated ALT, AST, creatine kinase (CK), creatine phosphokinase isoenzymes (CPK), lactic dehydrogenase (LDH), and plasma glucose concentration, and decreased albumin levels were observed in more than half of cases at admission, and developed in nearly all cases during hospitalization. Elevated creatine level was observed in 25% of cases during hospitalization. Seventeen (77%) cases developed proteinuria at a median of 9.0 days (IQR 7.0–11) after illness onset.

### Treatment

All cases received empiric treatment with broad-spectrum antibiotics during hospitalization, including ceftriaxone (n = 6), moxifloxacin (n = 8) and azithromycin (n = 15). Corticosteroids (median methylprednisolone dosage 1.6 [1]–[5] mg/kg per day IV) were initiated at a median of 6.5 days (IQR 6.0–8.0) from illness onset and administered to 24 (92%) cases for a median of 6 days (IQR 3–13). Of these, 21 (88%) received high-dose corticosteroids.

Four children received late antiviral treatment. One was treated with amantadine (100 mg per os (po) twice daily (BID) on illness days 10–15) and ribavirin (200 mg IV/d on illness days 9–16), and one received rimantadine (100 mg po each day (qD) on illness days 9–11); both cases survived. One child received oseltamivir (37.5 mg po BID) on illness days 12–14, and one child was treated with oseltamivir (40 mg po qD) on illness day 10; both died. Eight adults received late oseltamivir treatment, including two fatal cases – one received 75 mg po BID on illness days 8–11, and one received both oseltamivir (75mg po BID on illness days 11–20) and rimantadine (200mg po qD on illness day 11). Six adults treated with oseltamivir survived: one was treated with 75 mg/day on illness days 8–12, four received 75 mg BID on illness days 4–11, illness days 8–14, illness days 10–14, and illness days 8–12, respectively, and one was treated with 75mg BID and amantadine (100 mg po BID) on illness days 8–12.

Two critically ill adult H5N1 cases (31-year-old male, 44-year-old female) with ARDS were treated with convalescent plasma obtained from one of two fully recovered H5N1 adult donor cases. Plasma was obtained 129 days after illness onset from an adult female case and 81 days after illness onset from an adult male case. Both donors' convalescent plasma tested negative for hepatitis B, hepatitis C, and HIV, and were separated and heat-inactivated at 56°C for 10 h before transfusion. The male ARDS case received three units (200 mL/unit) of transfused convalescent plasma from the female donor for 2 days, beginning on illness day 13. His H5N1 viral titre in bronchial-alveolar lavage fluid declined substantially and was undetectable for the next 3 consecutive days after receipt of the third convalescent plasma dose. The female ARDS case, who had a history of bronchiectasis, received one unit (200 mL) of transfused convalescent plasma from the male donor once daily for 3 days, starting on illness day 13. Further virological testing has not been done for this case. Both cases also received oseltamivir (75 mg po BID) on illness days 10–14 and days 8–12, respectively. Both cases recovered fully and were discharged home.

### Complications and outcomes

Twenty-three (88%) cases required ventilatory support for respiratory failure. ARDS developed in 21 (81%) cases at a median of 8 days (IQR 7–9) after illness onset. Liver function impairment, renal dysfunction and cardiac failure occurred in 9 (43%), 4 (17%) and 13 (50%) patients.

Seventeen (65%) cases died (2 children, 1 adolescent and 14 adults), including one pregnant woman at 4 months' gestation [28] after a median of 10 days (IQR 8–20.5). Nine (35%) nonfatal cases were discharged at a median of 41 days (IQR 31.5–64.0) after illness onset. Five (24%) of the 21 cases with ARDS survived, including one pregnant woman, two adults who received convalescent H5N1 plasma, and two other previously healthy adults. The pregnant woman survived after developing ARDS and experiencing a spontaneous abortion during mechanical ventilation. Her pulmonary status subsequently improved and her temperature normalised quickly; the patient was extubated and recovered completely. All 17 fatal cases had multi-organ failure, including respiratory failure (94%), cardiac failure (71%), renal failure (27%) and 24% had disseminated intravenous coagulation (Table 4).

**Table 4:** Comparison of demographic and clinical features of 17 fatal and 9 nonfatal H5N1 cases, China.

Variables	Fatal cases, n = 17	Survivors, n = 9	p-value*
Demographic features			
Male, no. (%)	7 (41)	4 (44)	1.000
Age, median (IQR)	29.0 (20.5–38.0)	26.0 (8.5–34.0)	0.403
Year			
Oct 2005–Sep 2006, no. (%)	13 (76)	7 (78)	0.315
Oct 2006–Sep 2007, no. (%)	1 (6)	2 (22)	
Oct 2007–Apr 2008, no. (%)	3 (18)	0 (0)	
H5N1 Virus clade			
Clade 2.2, no. (%)	1 (6)	1 (11)	1.000
Clade 2.3.4, no. (%)	16 (94)	8 (89)	
With underlying medical conditions,‡ no. (%)	2 (12)	2 (22)	0.591
Medical care			
Median time in days from illness onset to hospitalization (IQR)	6.0 (4.5–7.5)	8.0 (7.0–9.0)	0.053
Level of hospital admission, no. (%)			
County	7 (41)	1 (11)	0.276
Prefecture	6 (35)	6 (67)	
Province	4 (24)	2 (22)	
Haematological markers at admission			
Median WBC (×10^9^ cells per L) (IQR)	3.8 (2.7–5.0)	3.3 (1.7–3.8)	0.196
Median LYM (×10^9^ cells per L) (IQR)	0.5 (0.4–1.1)	0.7 (0.4–1.1)	0.571
Median PLT (×10^9^ per L) (IQR)	78 (62.0–121.5)	99.0 (74.5–123.0)	0.332
Nadir measurement of haematological markers during hospitalisation			
Median WBC (×10^9^ cells per L) (IQR)	2.0 (1.3–2.9) [16] ¶	2.6 (1.7–2.8)	0.522
Median LYM (×10^9^ cells per L) (IQR)	0.4 (0.3–0.5) [16]	0.3 (0.3–0.5) [7]	0.922
Median PLT (×10^9^ per L) (IQR)	64.5 (34.5–75.0) [14]	93.0 (56.0–99.0) [7]	0.020
Biochemical markers at admission			
Median ALT (U/L) (IQR)	103.0 (24.0–182.0) [15]	31.0 (18.0–50.5)	0.079
Median AST (U/L) (IQR)	168.0 (72.0–513.0) [15]	94.0 (52.5–276.5)	0.245
Median Albumin (g/L) (IQR)	29.2 (27.1–34.3) [14]	30.7 (28.7–38.6) [8]	0.339
Median CK (U/L) (IQR)	1167.5 (191.5–1420.2) [12]	597.5 (292.5–3932.8) [8]	0.792
Median CK-MB (U/L) (IQR)	66.5 (23.0–127.1) [10]	27.0 (5.0–42.0) [7]	0.161
Median LDH (U/L) (IQR)	1860.0 (796.0–2272.0) [11]	1230.0 (551.5–1549.5)	0.230
Peak measurement of biochemical markers during hospitalisation			
Median ALT (U/L) (IQR)	183.0 (103.0–224.0) [13]	178.0 (78.3–458.0) [6]	1.000
Median AST (U/L) (IQR)	427.0 (165.0–734.0) [15]	159.0 (102.0–276.5)	0.084
Median Albumin (g/L) (IQR)	27.1 (24.1–29.8) [13]	30.0 (26.3–30.6) [7]	0.393
Median CK (U/L) (IQR)	1914.5 (846.8–9358.3) [12]	676.0 (537.0–4880.0)[7]	0.142
Median CK-MB (U/L) (IQR)	83.0 (72.0–282.1) [9]	74.0 (42.5–3455.0) [5]	0.606
Median LDH (U/L) (IQR)	1982.5 (1764.8–2978.3) [12]	1230.0 (1104.5–1702.0)	0.001
Complications			
ARDS	16 (94)	5 (56)	0.034
Median time in days from illness onset to ARDS (IQR)	7.0 (7.0–8.8) [16]	8.0 (8.0–12.0) [5]	0.075
Respiratory failure	16 (94)	7 (78)	0.268
Liver function impairment	6/14 (43)	3/7 (43)	1.000
Renal dysfunction	4/15 (27)	0/9 (0)	0.259
Cardiac failure	12 (71)	1 (11)	0.011
DIC	4 (24)	1 (11)	0.628
Treatments			
Antiviral therapy#			
No antiviral treatment	13 (76)	1 (11)	P = 0.003, with a positive correlation#
Received any antiviral treatment	4 (24)	8 (89)	
Amantadine or rimantadine only	0 (0)	3 (33)	
Oseltamivir only	3 (18)	5 (56)	
Combined amantadine (rimantadine) and oseltamivir	1 (6)	0 (0)	
Median time in days from illness onset to initiation of antiviral therapy (IQR)	12.0 (8.5–11.8) [4]	8.0 (8.0–9.8) [8]	0.109
Administered high-dose corticosteroids$	14/15 (93)	7 (78)	0.533
Median time in days from illness onset to initiation of corticosteroids (IQR)	7.0 (6.0–10.0) [15]	8.0 (7.5–10.0)	0.174
Median days of corticosteroid therapy (IQR)	4.0 (2.0–9.0) [15]	12.0 (6.0–16.5)	0.025
Plasma therapy	0 (0)	2 (22)	0.111

* Medians were compared between fatal and survival cases with the Wilcoxon rank sum test. For categorical variables, percentages of cases in each category were compared with Fisher's exact test.

† NA demotes not applicable.

‡ Two fatal H5N1 cases had underlying medical conditions, including a 24-year-old pregnant woman [28] and a 16-year-old male with a 10-year history of minimal change glomerulopathy. Two surviving H5N1 cases had underlying medical conditions, including a 26-year-old pregnant woman and a 44-year-old female with a ten-year history of chronic bronchitis [unpublished data, China CDC].

# A higher proportion of cases survived that received any antiviral treatment compared to those that did not receive antivirals (67% [8/12 patients] vs 7% [1/14 patients], p = 0.003), and with a positive linear association: the Gamma coefficient equals 0.664 (p = 0.005) which indicate a positive correlation between antiviral therapy and disease outcome.

$ High-dose corticosteroid use was defined as ≥250 mg hydrocortisone or equivalent intravenous (IV) administration daily. For children <13 years old, high-dose corticosteroid use was defined as ≥5 mg hydrocortisone or equivalent IV/kg/day.

¶ []: Indicates denominators for testing of fewer cases than full group.

In the bivariate analyses, demographic characteristics, year of illness onset, clade of H5N1 virus infection, and underlying medical conditions were similar between fatal and nonfatal cases (Table 4). Fatal cases had significantly lower median nadir platelet count during hospitalization (64.5×10^9^ cells/L *vs* 93.0×10^9^ cells/L, p = 0.02), higher median peak LDH level during hospitalization (1982.5 U/L *vs* 1230.0 U/L, p = 0.001), higher frequency of ARDS (94% [n = 16] *vs* 56% [5], p = 0.034), more frequent cardiac failure (71% [n = 12] *vs* 11% [1], p = 0.011), and shorter median duration of corticosteroid therapy (4.0 days *vs* 12.0 days, p = 0.025) compared to cases that survived. A higher proportion of cases survived that received any antiviral treatment compared to those that did not receive antivirals (67% [8/12 patients] *vs* 7% [1/14 patients], p = 0.003), with a positive correlation between antiviral therapy and disease outcome (Gamma coefficient = 0.664, p = 0.005).

## Discussion

Our findings suggest that H5N1 disease in Chinese patients generally begins with fever, cough, and sputum production, and progresses rapidly to lower respiratory disease. Upper respiratory symptoms of rhinorrhea and sore throat were less common in China than observed in Hong Kong SAR, China [9], Thailand [13], Turkey [21], Azerbaijan [18], and Egypt [2]. Studies suggest that the lower respiratory tract is the major site for H5N1 viral replication, although initial infection may occur in either the upper or lower respiratory tract [30]–[33].

Diarrhea was present in only two H5N1 cases at admission, but developed in a quarter of cases during hospitalization. Diarrhea was a common presenting symptom among H5N1 cases in Vietnam [11], [12] and Thailand [13], but was reported infrequently among cases in Hong Kong SAR, China [9], [10], and Indonesia [4], [16]. H5N1 virus and viral RNA have been detected in feces and intestines of human H5N1 cases [12], [17], [30], [33]. Whether the gastrointestinal tract is a primary site for H5N1 virus infection is currently unknown.

Disease course in Chinese H5N1 cases was rapidly progressive; the median time from illness onset to death in our case series is consistent with WHO findings [2]. All H5N1 cases presented with pulmonary infiltrates, and all cases progressed rapidly to bilateral disease. Many cases experienced respiratory failure, ARDS, and multi-organ failure, with hepatic dysfunction and cardiac failure. Leukopenia and lymphopenia were also common. A recent molecular pathology study on two cases documented that in addition to the lungs, H5N1 virus infects the trachea and disseminates to other organs including the brain [30]. Our findings are consistent with other reports [11]–[20]. The pathogenesis of some clinical complications could be immunologically mediated, as suggested by high levels of proinflammatory cytokines and chemokines in vitro and cytokine dysregulation in fatal cases in observational studies [10], [33], [34].

Five H5N1 cases were younger than 10 years old and one was aged 16-years, in contrast to other case series [16], [19]–[20] and the WHO finding that the highest frequency of cases was aged 10–19 years old [35]. The age profile of Chinese H5N1 cases may reflect exposure differences due to traditional social and cultural behaviours. Visiting wet poultry markets in urban areas and exposure to sick or dead backyard poultry in rural areas before illness onset are H5N1 risk factors in China (unpublished data, China CDC). Paediatric cases lived in rural areas of China, and likely had more exposures to sick/dead backyard poultry than children in urban areas. In rural areas, young Chinese children are much more likely to play with backyard poultry than older children. Adults are much more likely to visit poultry markets in urban areas of China than children and all urban adult H5N1 cases had visited a wet poultry market prior to illness onset (unpublished data, China CDC).

In contrast to the WHO finding that cases aged 10–19 years old had the highest case-fatality [2], mortality of H5N1 cases in China was not associated with median age, sex or underlying medical conditions in the bivariate analysis. Isolates from 24 cases in southern China were characterized as H5N1 clade 2.3.4 viruses with consistent genetic and antigenic properties from 2005 through 2008 (unpublished data, China CDC). There were no significant differences in case-fatality ratios between years during 2005–2008 or between cases with clade 2.2 and clade 2.3.4 H5N1 virus infection. However, fatal outcomes were associated with decreased platelet counts, increased LDH, ARDS, cardiac failure, and lack of antiviral treatment in the bivariate analyses. In Thailand [13] and Hong Kong SAR [9], mortality was associated with late presentation, lower admission leukocyte, platelet, and lymphocyte counts, bilateral pulmonary findings on chest X-ray, and development of ARDS. Decreased leukocyte and lymphocyte counts, and increased d-dimer levels were associated with fatal outcomes in other studies [4], [17], [20], [33].

Survival was significantly higher in cases that received any antiviral treatment than in untreated cases, and 5 of 8 adult cases that received standard oseltamivir treatment survived even though all were treated late in their illnesses. However, it should be noted that treatment was uncontrolled and our findings lack sequential virological data on antiviral susceptibilities or quantitative H5N1 viral shedding, and favorable outcomes and clinical courses of some H5N1 cases cannot be attributed definitively to antiviral treatment. In contrast to clade 1 H5N1 viruses isolated in Vietnam and clade 2.1 viruses in Indonesia [2], the clade 2.3.4 and clade 2.2 H5N1 viruses isolated from cases in China were susceptible to both M2 inhibitors and neuraminidase inhibitors (unpublished data, China CDC). These findings suggest roles for either class of antiviral drugs as well as combination antiviral therapy for H5N1 cases in China [36], [37].

Very few Chinese H5N1 cases received early antiviral treatment because only one patient was admitted within two days of illness onset, and no patients received outpatient antiviral treatment. Antivirals were not administrated to most Chinese H5N1 cases until they were hospitalized with pneumonia. Oseltamivir was not available in some hospitals for treatment of some cases that died. Therefore, education of health-care providers about the epidemiological risk factors and clinical characteristics of H5N1 patients, and wider availability of antiviral drugs could help facilitate earlier detection and treatment of H5N1 cases in China. Although little data on early versus late oseltamivir treatment for H5N1 patients are available, current WHO guidance recommends initiating oseltamivir treatment as early as possible, including consideration of higher dosing for severe disease and longer treatment duration because of prolonged viral replication [37].

Although antiviral therapy is the primary treatment, most clinical management of H5N1 disease is supportive. For severely ill Chinese H5N1 patients with ARDS or multiorgan failure, management has focused on appropriate mechanical ventilation, correction of hypoxemia, fluid management, and treatment of other complications such as DIC. Corticosteroids were administered empirically to most H5N1 cases in China. A reduction in the proportion of cases reporting with fever from illness onset (92%) to hospital admission (69%) may reflect an early use of corticosteroids or non steroidal anti-inflammatory drugs. Compared to fatal cases, nonfatal cases in China had a longer duration of corticosteroid treatment. However, we cannot conclude that corticosteroid therapy resulted in survival and such treatment has not been shown to be effective in H5N1 patients [2]. Furthermore, prolonged or high-dose corticosteroid therapy may result in serious adverse events, including infection with opportunistic pathogens. Recent WHO H5N1 treatment guidance recommends against routine use of corticosteroid treatment [37].

Two cases with ARDS survived after receiving passive immunotherapy with transfused convalescent plasma from surviving H5N1 cases. This is compelling, but since passive immunotherapy and other treatments were administered in an uncontrolled manner, no definitive conclusions can be made about the benefit of such treatment [38]. A third Chinese H5N1 case survived after receiving post-vaccination plasma from an H5N1 vaccine clinical trial participant and combination antiviral treatment [5]. A meta-analysis of studies of convalescent plasma treatment during the 1918 influenza pandemic [39], evidence from animal experiments [40]–[42], and the limited experience in three Chinese H5N1 cases suggest that passive immunotherapy may be a viable option for the treatment of H5N1. Further research is needed to investigate the efficacy and effectiveness of passive immunotherapy with H5N1 convalescent plasma treatment for H5N1 patients, including cases with severe complications such as ARDS.

Our study was limited to available data for H5N1 cases identified through surveillance during the study period. Due to the small number of H5N1 cases, the study was too underpowered to compare differences between fatal and nonfatal cases. National surveillance and laboratory testing might not have identified all H5N1 cases that occurred, especially if the cases were clinically mild. Clinical management was uncontrolled, H5N1 viral shedding data, immunological and pathological data were not available, and any differences in outcomes cannot be interpreted to be due to the use of antiviral drugs, corticosteroids, or other uncontrolled treatments.

To improve clinical management of H5N1 patients in China, physicians should be educated about the natural history of H5N1 disease and epidemiological risk factors, and therapy should be standardized based upon current knowledge [37]. Early antiviral treatment and expanded testing should be considered for suspected H5N1 patients, with wider availability of antiviral medications at all health care facilities. In the absence of any definitive treatment for H5N1, preventive education to reduce risk behaviours for H5N1 exposures (e.g. avoiding direct contact with sick or dead poultry) must be emphasized more strongly.
